# Supplementary figures and images for: Database size positively correlates with the loss of species-level taxonomic resolution for the 16S rRNA and other prokaryotic marker genes
Source: PLoS Comput Biol. 2024 Aug 5;20(8):e1012343. doi: 10.1371/journal.pcbi.1012343 (PMC11326629; doi:10.1371/journal.pcbi.1012343)

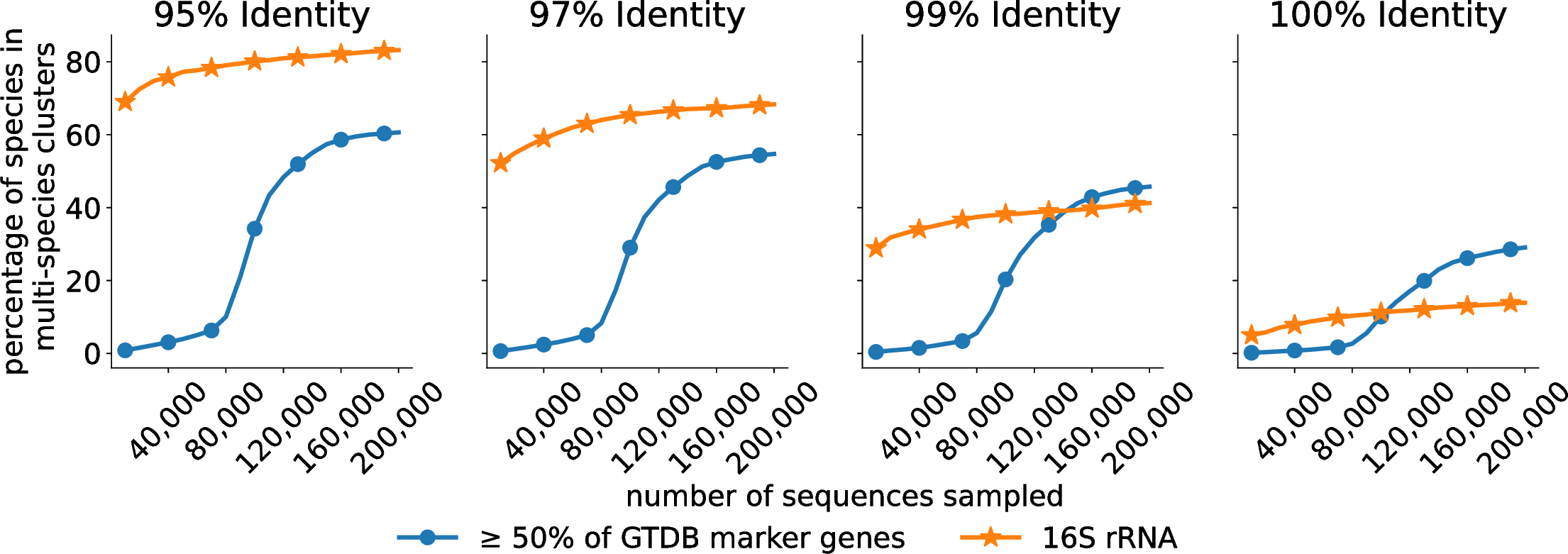

Supplement: S1 Fig — The percentage of species in multi-species clusters is also plotted for the 16S rRNA gene (denoted by a star in all subplots). The simulated databases were created by randomly sampling sequences from the 16S rRNA SILVA database and from the 120 genes used by the Genome Taxonomy Database (GTDB). Each simulated database was clustered with CD-Hit at several sequence identity cut-offs (95%, 97%, 99%, 100%), requiring that shorter sequences fully align to longer ones. (TIF) [file pcbi.1012343.s001.tif]
