# Supplementary material for: Database size positively correlates with the loss of species-level taxonomic resolution for the 16S rRNA and other prokaryotic marker genes
Source: PLoS Comput Biol. 2024 Aug 5;20(8):e1012343. doi: 10.1371/journal.pcbi.1012343 (PMC11326629; doi:10.1371/journal.pcbi.1012343)
Supplement: Attachment — Submitted filename: Response to PLOS Computational Biology Reviewers.docx [file pcbi.1012343.s004.docx]

# Reviewer #1

## Major comments

The authors did not demonstrate that database size is a problem to existing taxonomic classifiers based on marker genes. While it would be an important task to test how database size affects accuracy of taxonomic classification, this was not done in this paper.

We agree with the reviewer that we did not specifically test any taxonomic classification tools and acknowledge. Our purpose was to analyze the underlying data used by tools to provide more generalizable results. However, we acknowledge that the Background did not sufficiently provide a context for our work. As such, we have updated the manuscript to incorporate these clarifications and justifications from the existing literature.

On line 79, the authors state that ‘Multi-species’ clusters approximate the likelihood that a classifier would be unable to distinguish between the distinct species found’, suggesting that this could be a proxy for how accurate taxonomic classification would be. However, the authors provide no evidence to support this.

We have updated the manuscript to provide further justification for this claim.

As mentioned above, no classifier relies on a single marker gene for classification, and thus analysis at per gene level does not address the question.

We have added further discussion on this point. While many tools purport to use multiple marker genes or genomic regions, the actual classification is frequently still performed on the basis of markers considered in isolation from each other.

In addition, this completely disregards the algorithms behind different classifiers that might or might not be affected differently by databases of different sizes. In order to show that database size affects taxonomic classification accuracy, the authors would have to create databases of varying sizes for a set of commonly used taxonomic classifiers (see above) and use a ‘ground truth’ dataset to evaluate classification accuracy. Otherwise, it appears that they are calling attention to a problem that does not yet exist.

We agree with the reviewer that we did not specifically test any taxonomic classification algorithms and acknowledge that the Background did not sufficiently provide a context for our work. However, we believe we can justify that the experiments suggested by the reviewer are not necessary. There is abundant literature about the negative effects of redundancy in databases, and multi-mapped reads on taxonomic classification. Further, there are many tools that acknowledge the issue and explicitly attempt to handle it. We added some of these points and citations to the manuscript to clarify this issue and to provide context for our analysis. Here are some references that highlight these issues.

1) Rapid and precise alignment of raw reads against redundant databases with KMA
2) A critical assessment of gene catalogs for metagenomic analysis
3) Embracing Ambiguity in the Taxonomic Classification of Microbiome Sequencing Data
4) Metalign: efficient alignment-based metagenomic profiling via containment min hash
5) AFS: identification and quantification of species composition by metagenomic sequencing

We do want to note that despite some appreciation for these issues in the bioinformatics community, approaches based on single markers continue to be the most widely used in practice, and our manuscript intends to further caution against such widely-held misconceptions.

The metrics used in the study are hard to interpret. For example, for figures 1A and 2A it is hard to assess importance/prevalence of the multi-species cluster without knowing the total numbers of clusters, these graphs might be more informative as a proportion.

We have added this information to the figures to increase interpretability.

Similarly, for figure 1C, since the tools do not rely on a single gene, % of species with at least one gene in multi-species cluster is not very informative. More informative could be % of species with all or at least majority of marker genes in multi-species clusters.

We have added a new figure, Figure 3, that highlights the percentage of species with at least 50 marker genes in multispecies clusters.

I could not find a link to the code used for the analysis of the data.

We created a GitHub page for the analysis here: <https://github.com/tluan/ProkaryoticMarkerGenes-DatabaseSizeAnalysis>

## Minor Comments

Line 69: The overlap should be more than 3 genes, it would be good to see how the mapping between GTDB and 40 marker genes was done.

The reviewer is correct. Originally, we had taken this number from a prior study, but we performed our own analysis which found 29 marker genes in the intersection. This has now been corrected in the manuscript.

Diagram of the workflow and a separate Methods section would be useful to assess the methods

We appreciate the reviewer’s recommendation. We created a workflow diagram and added a separate Methods section.

Choice of Listeria for the second part of analysis is unclear

We added clarification to the manuscript. The SILVA database and GTDB had shown the broad effect of database size on the loss of taxonomic resolution. The Listeria analysis was performed to assess if the same pattern can be seen for a deeply-sampled taxonomic group like Listeria, which contains a well-known pathogen.

Linear regressions in Figure 1D have 2 outliers/high leverage points (one of which seems to have been cut out of the second panel), these should either be done in log space or removed.

We appreciate the reviewer catching this. The outliers in Figure 1D (97% subplot) were cut out because the xlim parameter for plotting was too low. We increased the xlim to 1700 and the issue was fixed. We decided to keep the current scale since we are applying a linear regression model, and kept the outliers because they provide insight into the overall trend.

On line 150 the authors state that many tools rely on individual genes for classification at species level, I am not sure which ones they mean (the references 16 and 20 do not, and k-mer based tools are not relevant to the analysis performed). The authors’ conclusion is that a combination of genomic markers should be used (and that longer genomic segments are needed to differentiate between distinct species), is in fact what is done by GTDB and other tools relying on marker genes.

We’ve added clarification and citations to the manuscript (citations 6 to 22) that highlight approaches that assign taxonomic labels to individual metagenomic reads based upon mapping to individual genes.

# Reviewer #2

## Major questions

1. As this study pointed out, the previous marker genes, including 16S RNA genes don’t provide high-enough resolution to discriminate microbes at the species-level. Based on your analysis, are you able to identify new genes that can be served as marker genes for this classification purpose or some gene combination? Or maybe the concept of “marker gene” should be reconsidered?

We appreciate the reviewer’s question. The main conclusion from our analysis is that all individually tested marker genes are losing resolution, so carefully chosen marker combinations might be the way to go. Perhaps the complete set of 120 GTDB marker genes would be used, as they are currently highly discriminative. However, as highlighted in the Discussion, a study has shown that even the average nucleotide identity of all homologs in genomes might not be sufficient to differentiate all species. Another challenge for using all 120 GTDB marker genes is that there often isn’t enough sequencing depth to obtain coverage of all the marker genes for all taxa in metagenomic samples. We also discuss, as the reviewer suggested, that the concept of marker genes might need to be revisited. This might involve the combination of universal marker genes with clade-specific genes. However, we only highlight the issues here to help guide future research efforts.

2. The authors also mentioned that even 95% ANI may not serve as the species level cut-off. Please provide more details regarding the genome completeness of the study that you cited (ref 18, 21, 22) and >=100 genome sequences is not clear enough.

We added clarification to the manuscript, “highly sampled” species were defined as those with 100 or more sequenced genomes in NCBI. We are not able to provide detailed information about the completeness of the genomes used in that study as they did not provide that information. What they state in the methods is that two datasets were used: 1) 90,000 complete and draft assemblies (we’re not sure how many are draft versus complete); 2) 10,616 complete bacterial genomes downloaded from RefSeq.

ANI is quite a common idea for prokaryotes classification. I would suggest the author perform some analysis of some random genus to better show and support your results.

We appreciate the reviewer’s comment but left it as a Discussion point because other studies have already performed these analyses (cited in Discussion) and to do a thorough assessment would require an in-depth analysis which is beyond the scope of our current study. Also, we have plans to analyze this factor in future work.

3. There is another idea, called pangenome, where scientists tried to use core genes for species- or genus- level classification. Maybe incorporating this layer of information will better answer the question proposed in this study. You can refer to this data which may serve as a case study to support your research. <https://escholarship.org/uc/item/79t1w9dx>

Although an interesting area for future work, our current study was limited to the analysis of universally present marker genes in prokaryotes. In some sense, these marker genes can be thought of as the core genes of all prokaryotes. We agree that future work should expand upon our analysis and look at the trends in all gene families and the effect of a gene’s level of mobility.

## Minor questions

1. “For all genes, the number of sequences in multi-species clusters increased at a super-linear rate as the database grew (Figures 1A and 1B),” This sentence is not clear. “For all genes”, do you mean the 120 marker genes you used?

We added a section subheader under the Results to add clarity that the genes were from the SILVA database and the GTDB.

How do you define the “multi-species clusters”?

The definition is included in the Methods under the new subheader “Cluster analysis.”

2. “The rate at which sequences are clustered with sequences from other species was estimated using the Y = cXm 97 linear regression model in log-log space,” I think you can use similarity to describe how close the two sequences are. This makes readers easier to follow.

We’re not sure that we completely understand what the reviewer means. However, the sequence clustering accomplishes the reviewer’s goal of describing how closely related sequences are. The regression analysis was done to show that there is an increasing number of multi-species clusters, indicating a trend that databases will likely continue to lose species-level information as new sequences are added.

The Y-axis label of each subplot in Figure-1 is too close to the next figure, hard to tell is the title of which figure. The legend mentions that “the number of genes, the number of sequences in multi-species clusters, the total number of multi-species clusters…”, while in Figure 1, the X-axis label is “counts”, the Y-axis label is “number of sequences sampled”. The terminology you use in both legend and figures should be consistent. Otherwise, it is really hard to follow. Please check all figures and legend to address this issue.

We agree with the reviewer and updated the plots so their labels are standardized and the subplots are better separated to increase clarity.

# Reviewer #3

Overall I agree with the overall message of this manuscript, believe that it is sufficiently supported by the evidence presented, think this manuscript is a solid contribution to the field, and support its publication. Thoughts below are mostly about interpretation and language, and are offered as suggestions.

**It is not clear how well random sampling of these reference databases captures the longitudinal dynamics (i.e. database growth over the years) that it is intended to be simulating.** For example, the super-linear increase in sequences in multi-species clusters is necessarily true when subsampling in this way (at least averaged over subsampling realizations) since sequences are added to clusters at a uniform rate, and the number of clusters that are multi-species can only increase with size of the subsample. But the real growth in time of databases may not be the same as subsampling (where all re-orderings are equal, i.e. database entries are exchangeable). For example, the discovery of a new set of species that are well-separated from others could lead to a decrease in the fraction of sequences in multi-species clusters, that is not possible in the subsampling realization of database growth. Other dynamics (re-namings, splitting previously named species into multiple species etc.) also lead to entries that are not exchangeable with those that occurred before that event.

A limitation for our study is that we don’t have access to the previous versions of all the databases used for analysis. Nonetheless, a previous work that we cited (“RefSeq database growth influences the accuracy of k-mer-based lowest common ancestor species identification”) performed a longitudinal analysis of the RefSeq database versions and observed the same trends that we do.

We argue that a super-linear increase in sequences in multi-species clusters is not predictable, as the reviewer suggests. For example, there is a debate in the field about whether bacterial genetic diversity is discrete or continuous. If bacterial genetic diversity is discrete, as suggested by ANI genome clustering experiments, it might be expected that few gene-length interspecies sequence collisions occur.

The point that “the discovery of a new set of species that are well-separated from others could lead to a decrease in the fraction of sequences in multi-species clusters” is countered by our observation (Figure 1D) that the more highly sampled a genus is, the higher the number of multi-species clusters those sequences are members of. As this was a general trend for all genera in the SILVA and GTDB database, the trend would predict a similar result for new sets of species.

With respect to the reviewer’s point about the effect of “re-namings, splitting previously named species into multiple species,” it is uncertain how to account for this issue in a simulation given that the current taxonomy might not be correct or complete either and, therefore, cannot be treated as a ground truth. However, we did observe the same trends of loss of species resolution in the SILVA and GTDB databases, which use different taxonomies. On all these points, we are open to the reviewer’s suggestions.

L61-62; L107-109; L130-131: All three frame the observation that “database growth is affecting our ability to discriminate important pathogens from their near neighbors.” as a degradation in species (or pathogen) resolution. I’m not sure I agree with that framing. I agree that the likelihood of single named-species being output as the query within some %ID threshold from the reference database does decrease. But previously that was a mistake due to an incomplete reference database. The change in performance seems to me an improvement, not a degradation.

We agree with the author that understanding that some pathogens are highly similar to non-pathogenic species is an important increase in our knowledge and provides information to improve diagnostics. We updated our framing to reflect that while database growth is affecting our ability to discriminate important pathogens from their near neighbors with the tested marker genes, it suggests that pathogen classification should incorporate other genomic markers that contribute to pathogenesis and virulence e.g., toxin genes. The former point is still relevant because many tool users in the community take the output of classification tools at face value, even when used for pathogen detection in metagenomic datasets. Our analysis is a cautionary reminder that the output of classification tools is database-dependent, and the resolution might be decreasing.

L88-89: “Further, genes from distinct species that are 100% identical indicate that species level resolution is simply not possible for a specific gene.” I don’t agree with this. It shows there is a counter-example for that gene in which it is not possible to discriminate between a pair of species. That is, species resolution is not ALWAYS possible for that specific gene. But it may still be that that species-level resolution is often possible, e.g. Fig 1C 100% panel – ~20% of species are in multi-species cluster, but then 80% aren’t and can be speciated with a given gene. So species-resolution is possible with that gene most of the time. **This logic of a counter-example meaning species resolution is “impossible” appears in other places in the manuscript as well (and repeatedly in the literature).**

This is an interesting point by the reviewer; however, our analysis provides evidence that our observations do not merely identify counterexamples. Rather, our analysis shows growing trends both for the number of multi-species clusters and the number of species with membership in multi-species clusters. These trends were especially evident for highly-sampled genera with many species. These observations support our hypothesis that as databases grow, they will likely experience a persistent and/or intensifying decline in species-level resolution. Through this work, we aim to raise awareness about this phenomenon.

Figures are so low-resolution as to be nearly illegible.

We redrew the figures and provided as *.tiff and *.png with dpi=300.
